# Polycomb protein RING1A limits hematopoietic differentiation in myelodysplastic syndromes

**DOI:** 10.18632/oncotarget.22839

**Published:** 2017-12-01

**Authors:** Anna Palau, Anne-Kathrin Garz, Jeannine Diesch, Anabel Zwick, Roberto Malinverni, Vanesa Valero, Katrina Lappin, Raquel Casquero, Andreas Lennartsson, Johannes Zuber, Tomàs Navarro, Ken I. Mills, Katharina S. Götze, Marcus Buschbeck

**Affiliations:** ^1^ Josep Carreras Leukaemia Research Institute, Campus ICO-Germans Trias i Pujol, Universitat Autònoma de Barcelona, Badalona, Spain; ^2^ Program for Predictive and Personalized Medicine of Cancer, Germans Trias i Pujol Research Institute (PMPPC-IGTP), Badalona, Spain; ^3^ Department of Medicine III, Klinikum rechts der Isar, Technische Universität München, Munich, Germany; ^4^ German Cancer Consortium (DKTK) and German Cancer Research Center (DKFZ), Heidelberg, Germany; ^5^ Centre for Cancer Research and Cell Biology, Queen’s University Belfast, Belfast, United Kingdom; ^6^ Current address: Department of Biosciences and Nutrition, Karolinska Institutet, Stockholm, Sweden; ^7^ Research Institute of Molecular Pathology (IMP), Vienna Biocenter (VBC), Vienna, Austria; ^8^ Clinical Hematology Department, ICO-Hospital GermansTrias i Pujol, Universitat Autònoma de Barcelona, Badalona, Spain

**Keywords:** myelodysplastic syndromes, polycomb repressive complexes, epigenetic regulation, hematopoietic stem cells, cellular differentiation

## Abstract

Genetic lesions affecting epigenetic regulators are frequent in myelodysplastic syndromes (MDS). Polycomb proteins are key epigenetic regulators of differentiation and stemness that act as two multimeric complexes termed polycomb repressive complexes 1 and 2, PRC1 and PRC2, respectively. While components and regulators of PRC2 such as ASXL1 and EZH2 are frequently mutated in MDS and AML, little is known about the role of PRC1.

To analyze the role of PRC1, we have taken a functional approach testing PRC1 components in loss- and gain-of-function experiments that we found overexpressed in advanced MDS patients or dynamically expressed during normal hematopoiesis.

This approach allowed us to identify the enzymatically active component RING1A as the key PRC1 component in hematopoietic stem cells and MDS. Specifically, we found that RING1A is expressed in CD34^+^ bone marrow progenitor cells and further overexpressed in high-risk MDS patients. Knockdown of RING1A in an MDS-derived AML cell line facilitated spontaneous and retinoic acid-induced differentiation. Similarly, inactivation of RING1A in primary CD34^+^ cells augmented erythroid differentiation. Treatment with a small compound RING1 inhibitor reduced the colony forming capacity of CD34^+^ cells from MDS patients and healthy controls. In MDS patients higher RING1A expression associated with an increased number of dysplastic lineages and blasts. Our data suggests that RING1A is deregulated in MDS and plays a role in the erythroid development defect.

## INTRODUCTION

Myelodysplastic syndromes (MDS) are a heterogeneous group of clonal hematopoietic stem cell (HSC) disorders with a strong predisposition to acute myeloid leukemia (AML). MDS imply a defect of hematopoietic differentiation characterized by clinically apparent dysplasia in the bone marrow and cytopenias in the peripheral blood [1]. Based on the number and type of affected lineages, the number of dysplastic cells and undifferentiated blasts and the presence of frequent cytogenetic alterations, the world health organization (WHO) classifies MDS into several distinct subgroups [2]. The international prognostic scoring system (IPSS) and its revised version (IPSS-R) further incorporate cytogenetic information to stratify patients into risk groups with regard to prognosis (estimated overall survival and risk of progression to AML) [3, 4]. For the purpose of treatment strategy decisions, IPSS-R intermediate, high and very high are commonly summarized as higher-risk MDS. Once considered a rare disorder in the general population, MDS incidences are sharply rising with age making MDS the most frequent hematopoietic disorder in the elderly [5]. Secondary MDS can also arise in younger and older patients after aggressive cancer treatments with radiation or chemotherapy [6].

Current treatment options for MDS are limited. The only curative treatment for MDS patients is allogeneic HSC transplantation, which is not an option for many patients due to the high mortality associated with advanced age or the lack of suitable donors [6]. The immunomodulatory drug lenalidomide is effective in the low-risk subgroup of patients presenting an isolated loss of the long arm of chromosome 5, MDS del(5q) [7]. The DNA hypomethylating agent azacitidine is currently the standard of care for higher-risk MDS patients, but only 50% of treated patients show hematological improvements and a complete response is limited to as few as 10% to 15% (reviewed in [8]). Responses are transient with a median duration of 24 months and virtually all patients eventually relapse [9].

The heterogeneity of MDS, the frequent occurrence of age-related comorbidities and the limited treatment options make the management of MDS challenging. The development of novel targeted treatments based on a better understanding of the molecular pathogenesis of MDS is an unmet need.

Epigenetics is defined as the information that can be transmitted through the cell cycle independent of DNA sequence. Polycomb proteins are key epigenetic regulators of differentiation and stemness and frequently deregulated in cancer [10]. On the molecular level Polycomb proteins act as two multimeric complexes termed Polycomb repressive complexes 1 and 2, PRC1 and PRC2, respectively. The general dogma posits that PRC2 and PRC1 act in a hierarchical and sequential manner. First, PRC2 trimethylates histone H3 on Lysine 27 (H3K27me3) [11]. Second, this mark is recognized by PRC1 that catalyzes the monoubiquitination of histone H2A and mediates chromatin compaction and gene repression [12, 13].

The composition of PRC1 is much more variable than PRC2. Although PRC1 is composed of only four protein subunits, it is highly modular and 16 in part mutually exclusive components can hypothetically form 180 different complexes [14]. The catalytically active RING1 subunit is either RING1A or RING1B, which forms the core complex by binding one of six Polycomb group of ring finger (PCGF) proteins. One of five possible CBX proteins provides the affinity for H3K27me3 and pairs with one of three PHC proteins. Non-canonical PRC1 complexes contain RYBP or its homolog YAF2 instead of the CBX subunit and bind chromatin independently of H3K27me3 [15, 16].

MDS is caused by an age-dependent accumulation of genetic and epigenetic alterations in the HSC compartment [6, 17]. Gene lesions such as mutations, copy number changes or losses of heterozygosity have been detected in up to 95% of patients [5]. Genome-sequencing efforts of the last years have indicated that mutations affecting the epigenetic machinery are frequent in MDS [18, 19] with 45% of all MDS patients harboring at least one mutation in an epigenetic regulator [5]. In particular, inactivating mutations in genes encoding components and regulators of PRC2 such as EZH2 and ASXL1 are particularly frequent [17, 20, 21]. In contrast, no mutations have been found in PRC1 components.

Here, we have systematically analyzed the expression of all genes encoding components of PRC1 complexes and functionally studied a panel of PRC1 components that were overexpressed in MDS or dynamically expressed during normal hematopoiesis. This approach allowed us to identify RING1A as key PRC1 component in hematopoietic stem/progenitor cells (HSPC) and motivated us to evaluate the potential of RING1A as drug target for the treatment of MDS.

## RESULTS

### PRC1 components are differentially expressed in higher-risk MDS and during differentiation

In order to characterize the function of PRC1 components in the pathogenesis of MDS, we analyzed the expression of genes encoding its components (for simplicity referred to as canonical PRC1 genes) in the hematopoietic stem/progenitor cell compartment of MDS patients and healthy controls and during normal hematopoiesis. In our analysis we have further included genes encoding components of PRC2 and proteins that were identified in non-canonical PRC1 complexes [16].

**Figure 1:**
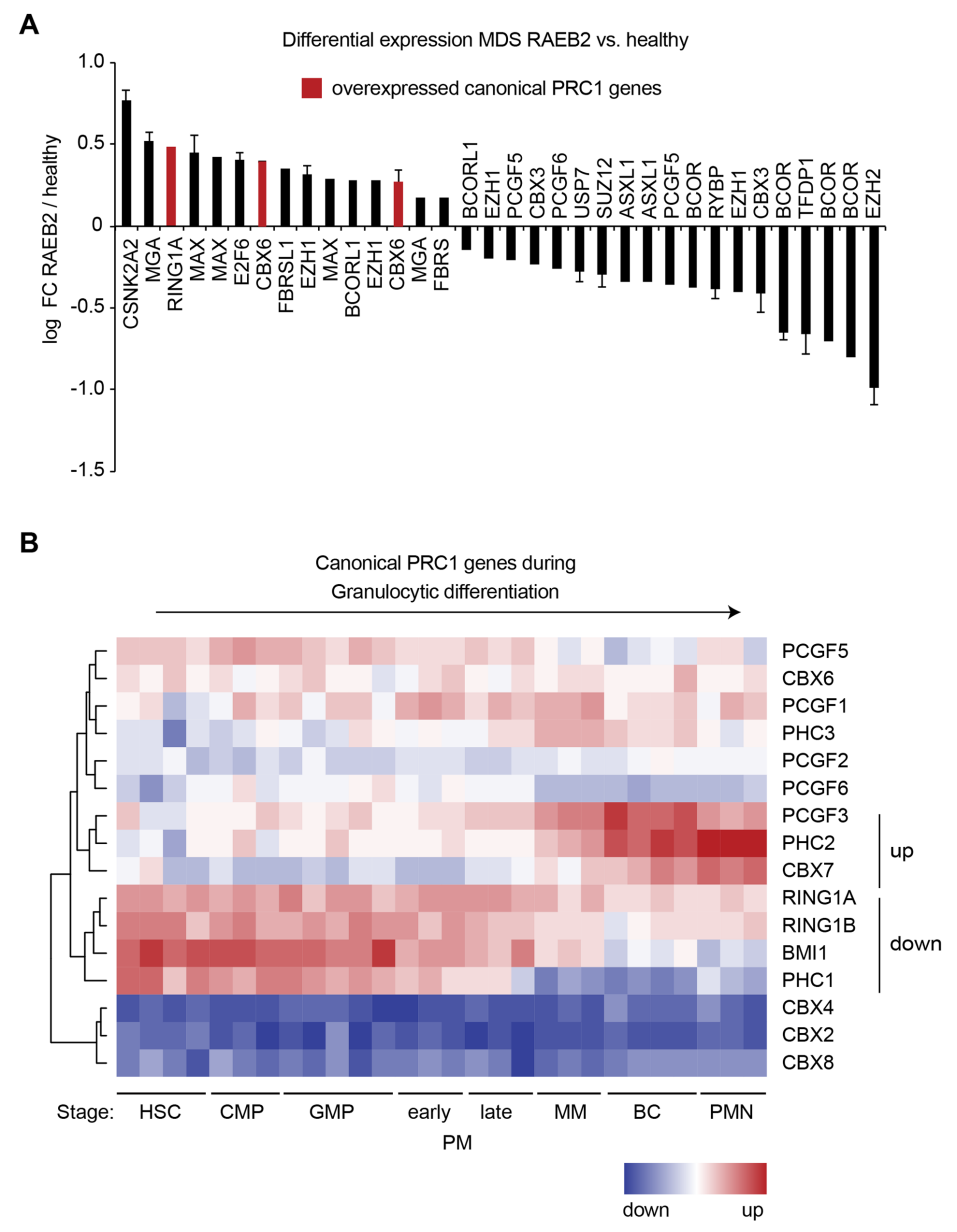
Expression analysis of PRC1 genes in MDS and differentiation **(A)** Logarithmic fold change in expression of probes for canonical PRC genes and components of non-canonical complexes in MDS classified as refractory anemia with excess blasts 2 (RAEB-2) compared to healthy controls. Two datasets [23, 24] were analyzed and only significant fold-changes (FC, p-value < 0.05) are shown. When significant in both datasets, the mean is plotted and the variation indicated by error bars. **(B)** Heatmap representing RNA expression of canonical PRC1 components during normal granulocytic differentiation [25]. Cell populations isolated from healthy bone marrow correspond to sequential steps in granulocytic differentiation that are hematopoietic stem cell (HSC), common myeloid progenitor (CMP), granulocyte-macrophage progenitor (GMP), early promyelocyte (early PM), late promyelocyte (late PM), metamyelocyte (MM), band cell (BC) and polymorphonuclear (PMN) mature granulocyte (n = 3-5). For all PRC1 genes see Supplementary Figure 2.

Long-term HSC (LT-HSC) are the most primitive HSC in a hierarchy of differentiation, with each subsequent cell type becoming more specialized [22]. LT-HSC as well as hematopoietic progenitors are enriched in the population of bone marrow cells expressing the CD34 surface marker [22]. In order to identify deregulations of individual genes, we used two datasets to compare the expression in CD34^+^ cells between healthy controls and high-risk MDS [23, 24]. These studies included MDS cases classified as refractory anemia with excess blasts 2 (RAEB-2) that correspond to MDS with excess blasts 2 (MDS-EB2) according to the revised 2017 WHO classification [2]. The genes most overexpressed in RAEB-2 included the canonical PRC1 components RING1A and CBX6 (Figure 1A). RING1A was also overexpressed in refractory anemia (RA) with ringed sideroblasts (RARS) but not in RA or RAEB-1 (Supplementary Figure 1A). Direct comparison of the probe intensities further suggested that RING1A is the predominantly expressed catalytic subunit of PRC1 (Supplementary Figure 1B). *EZH2* was the top downregulated gene in RAEB-2 (Figure 1A) and also scored significantly downregulated in the other MDS subtypes (Supplementary Figure 1A). Next, we were interested to understand to which extent the expression of PRC1 component encoding genes is dynamic during hematopoietic differentiation. For this we made use of an expression dataset of isolated bone marrow cell populations that represent eight sequential stages in the differentiation from HSC to fully mature polymorphonuclear granulocytes [25]. When focusing on canonical PRC1 genes, unsupervised hierarchical clustering divided the genes in four clusters (Figure 1B). The cluster of the most downregulated genes contained RING1A, RING1B, BMI1 and PHC1, while PCGF3, PHC2 and CBX7 were grouped together as those genes that were most upregulated during granulocytic differentiation (Figure 1B). In addition to these canonical PRC1 genes also many genes encoding components of the non-canonical PRC1 complexes were dynamically expressed during granulocytic differentiation (Supplementary Figure 2).

Taken together we have identified a subset of PRC1 genes that are highly expressed in the hematopoietic stem/progenitor compartment, overexpressed in MDS and dynamically regulated during granulocytic differentiation. Based on these results we have selected RING1A, BMI1, CBX6 and CBX7 for further analysis.

### Genetic perturbation studies in AML/MDS cells identify RING1A as key PRC1 component

**Figure 2:**
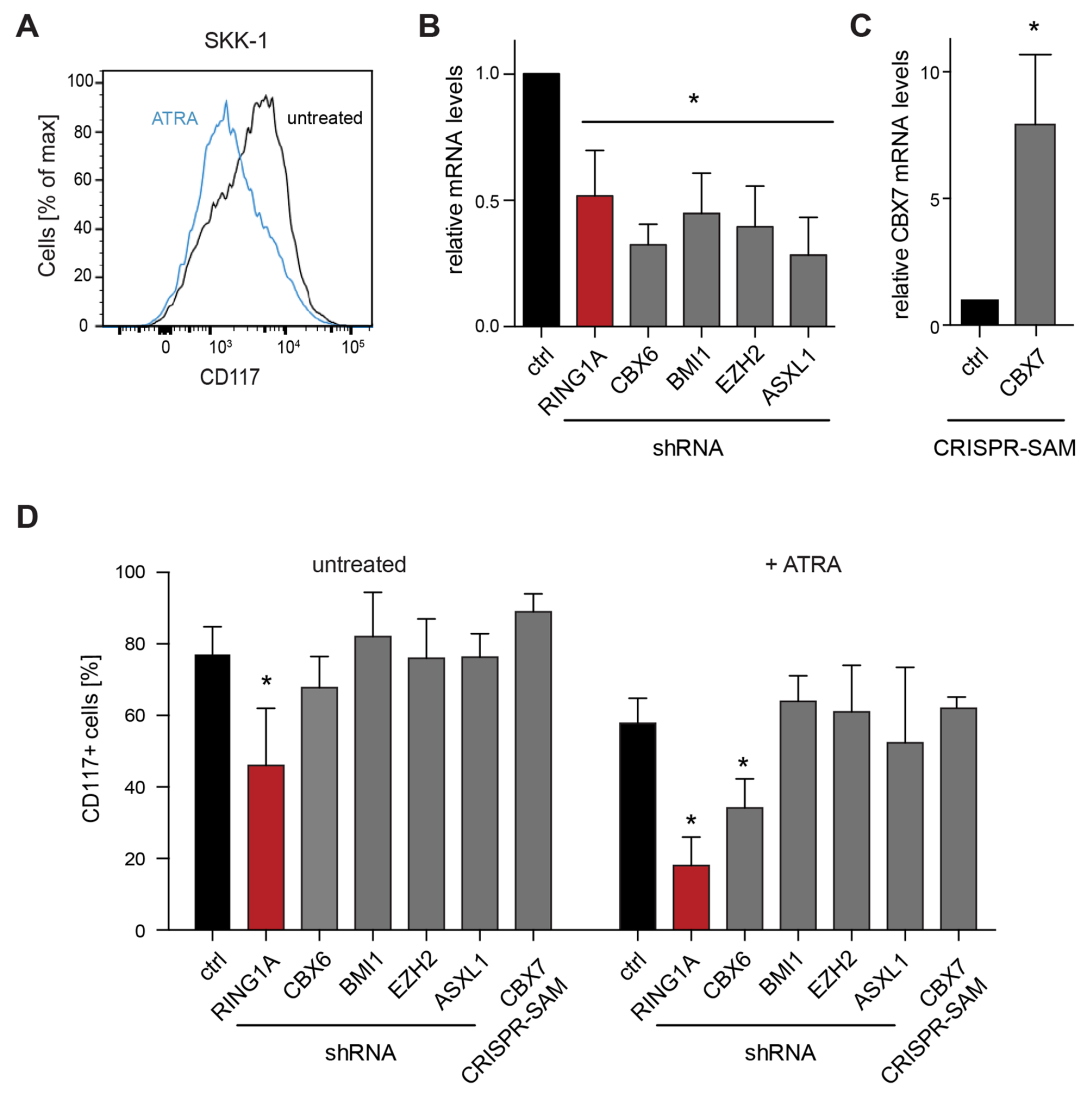
Genetic perturbation of PRC components in an MDS/AML cell line **(A)** SKK-1 cells respond to the treatment with 1μM ATRA by diminishing the levels of the pluripotency marker CD117, which was assessed by flow cytometry. **(B)** Knockdown efficiency of different shRNAs against PRC1 (RING1A, CBX6 and BMI1) and PRC2 (EZH2 and ASXL1) components. Data for CBX6, BMI1, EZH2 and ASXL1 shRNAs represents an average from two different shRNA sequences for each gene. The relative expression of the corresponding mRNA assessed by qRT-PCR compared to control cells (ctrl) was set to 1. Data is represented as mean + SD (n ≥ 4; ^*^, p<0.05 compared to control). **(C)** Overexpression of endogenous CBX7 using engineered CRISPR-Cas9 synergistic activation mediator (SAM). Data is plotted as in B. Data is represented as mean of three biological replicates + SD (^*^, p<0.05 compared to control). **(D)** Genetically perturbed SKK-1 cells shown in B and C were treated with ATRA as in A or left untreated. The percentage of CD117^+^ cells was assessed by flow cytometry. Data is represented as mean + SD (n ≥ 3; ^*^, p<0.05 compared to control).

MDS is characterized by defective hematopoietic differentiation. In order to test an influence of selected PRC1 components we decided to take a functional approach and studied the influence of genetic perturbations on the differentiation status and capacity of a model cell line. In a previous study we have characterized the immunophenotypes, cytogenetic and mutational profiles of a panel of MDS/AML cell lines that were derived from MDS patients after progression to AML [26]. For several reasons, we have selected the SKK-1 cell line as a suitable cell line to study the function of PRC1: First, SKK-1 cells express the pluripotency marker CD117 but are negative for most differentiation markers of the monocytic, granulocytic, megakaryocytic and erythroid lineages indicating their non-differentiated state. Second, SKK-1 cells have no mutations in the PRC2 components EZH2, EED, SUZ12 or its regulator ASXL1 [26]. Although SKK-1 cells have lost one copy of EZH2 [26], the remaining copy of EZH2 is wild-type and cells are positive for H3K27me3 [20], suggesting that the PRC2 complex is intact and functional. Third, we found that SKK-1 showed a partial response to the differentiation cue all-trans retinoic acid (ATRA) reflected in a reduction of the proportion of CD117^+^ cells as assessed by flow cytometry (Figure 2A). In terms of cytology, we observed a reduction in basophilia after May-Grünwald-Giemsa staining (Supplementary Figure 3A), a further characteristic of differentiation [27].

To evaluate the function of PRC1 genes, we used RNA interference to reduce the levels of RING1A, CBX6 and BMI1, which were highly expressed in CD34^+^ cells (Supplementary Figure 1B) and in the case of RING1A and CBX6 also overexpressed in MDS RAEB-2 (Figure 1A). As controls, we have included the PRC2 component EZH2 and the PRC2 regulator ASXL1. Using a lentiviral system and small hairpin RNAs (shRNAs) embedded in an optimized miRNA backbone [28], we achieved a significant reduction of the levels of the targeted mRNAs (Figure 2B). Furthermore, we decided to increase the expression of CBX7 that we found to be less expressed in CD34^+^ cells and further upregulated during differentiation (Supplementary Figure 1B and 1B). For this, we employed a modified CRISPR-Cas9 complex that allows the activation of the endogenous gene by RNA guided recruitment of transcriptional activators [29]. This technique allowed us to induce the expression of CBX7 approximately 8-fold (Figure 2C). Next we analyzed the influence of these manipulations on the differentiation state by reading out the number of cells displaying CD117 in steady-state growth conditions and after treatment with ATRA. Of all these genetic perturbations the knockdown of RING1A had the strongest influence and reduced the proportion of CD117^+^ cells and the overall expression of the CD117 before and after treatment with ATRA (Figure 2D and Supplementary Figure 3B). Knockdown of CBX6 had less effect in reducing the proportion of CD117^+^ cells only detectable by flow cytometry and after ATRA treatment (Figure 2D). Taken together, these results prompted us to focus our study on RING1A as a key candidate component of canonical PRC1 in MDS.

### The influence of RING1A on differentiation is isoform-specific

**Figure 3:**
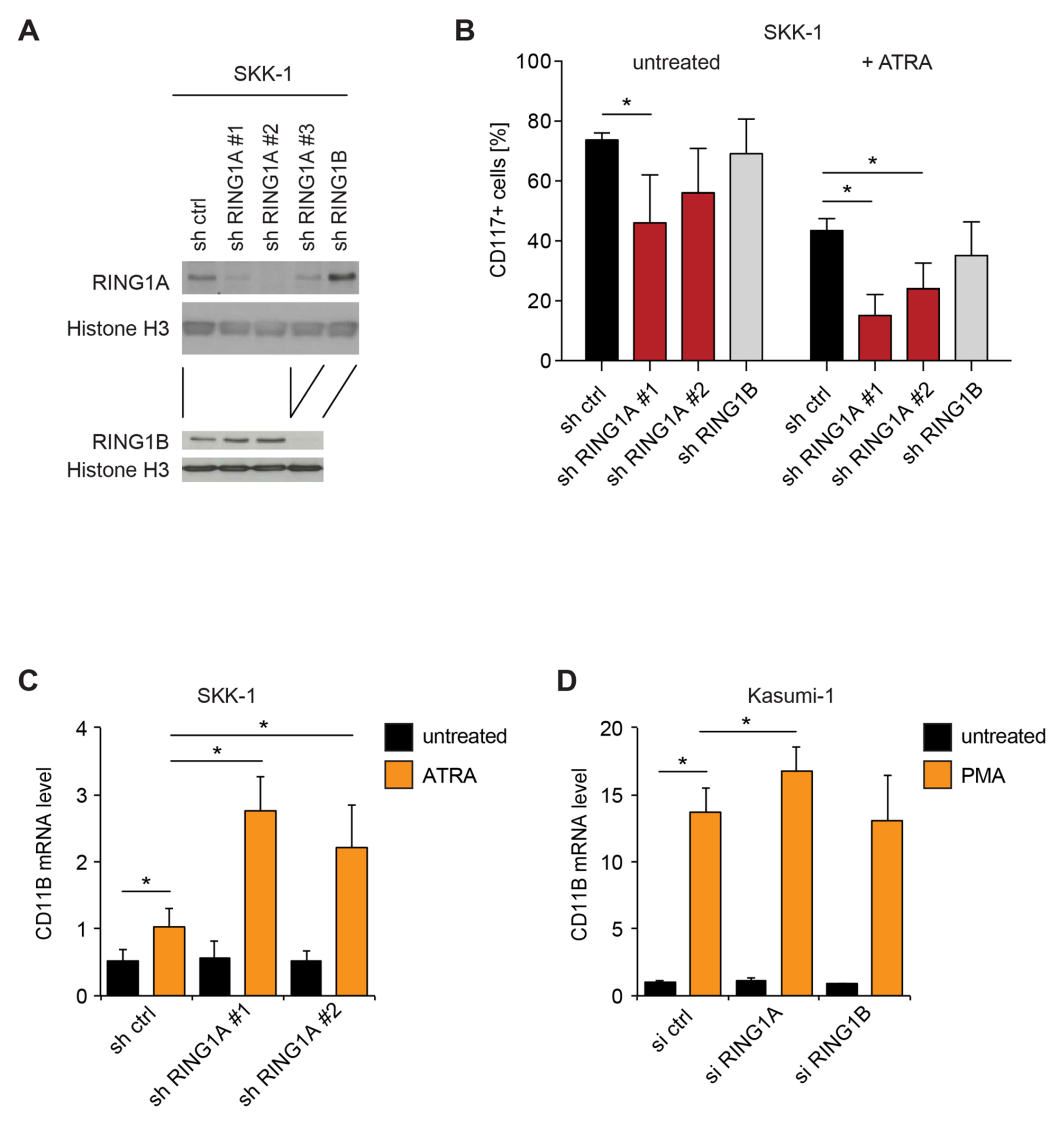
The influence of RING1A on differentiation is isoform-specific **(A)** Immunoblot analyses of RING1A and RING1B in total lysates of SKK-1 cells transduced with shRNA cassettes for RING1A, RING1B and a control hairpin (sh ctrl). An anti-Histone H3 immunoblot has been included to control for the protein content of samples. The shRNA against RING1A previously used (Figures 2 and Supplementary Figure 3) is here referred to as #1. **(B)** SKK-1 sh control (ctrl), shRING1A#1, shRING1A#2 and shRING1B#1 cells were treated or not with 1 μM ATRA for two days and the percentage of CD117^+^ cells was assessed by flow cytometry. Data is represented as mean + SD (n ≥ 4; ^*^, p < 0.05). **(C)** The mRNA level of CD11B was analyzed in infected SKK-1 cells after 4 days of ATRA treatment. qRT-PCR data is presented as mean + SD (n = 4; ^*^, p < 0.05). **(D)** The mRNA level of CD11B was analyzed in siRNA transfected Kasumi-1 cells treated with 200 nM phorbolmyristateacetate (PMA) for 24 hours or DMSO vehicle (untreated). qRT-PCR data is presented as mean + SD (n = 3; ^*^, p < 0.05).

In order to further validate our findings, we repeated the suppression of RING1A in SKK-1 cells with a different shRNA and further also included one shRNA against the closely related isoform RING1B. As shown in Figure 3A, both RING1A and RING1B proteins are easily detected in SKK-1 cells and their expression was efficiently suppressed in cells transduced with the targeting shRNA vectors (Figure 3A). Interestingly, the knockdown of either RING1A or RING1B led to an increase in the other protein. A possible explanation for this observation might be provided by reported negative feedback loops in which Polycomb complexes repress the expression of genes encoding other Polycomb proteins [30]. Importantly, when treating SKK-1 cells with ATRA both shRNAs directed against RING1A reduced the number of CD117^+^ cells, while the similarly efficient shRNA against RING1B had no effect (Figure 3B). Conversely, the mRNA level of the myelomonocytic differentiation marker CD11b was elevated in ATRA-treated SKK-1 cells and further increased in RING1A knockdown cells (Figure 3C). Similarly, CD11b mRNA was also increased in phorbolester-induced Kasumi-1 acute myeloid leukemia cells and this increase was higher when cells were transfected with a pool of specific siRNAs for RING1A but not RING1B (Figure 3D). Together these results further support a role for RING1A in limiting cellular differentiation.

### RING1A limits differentiation in the hematopoietic stem cell compartment

**Figure 4:**
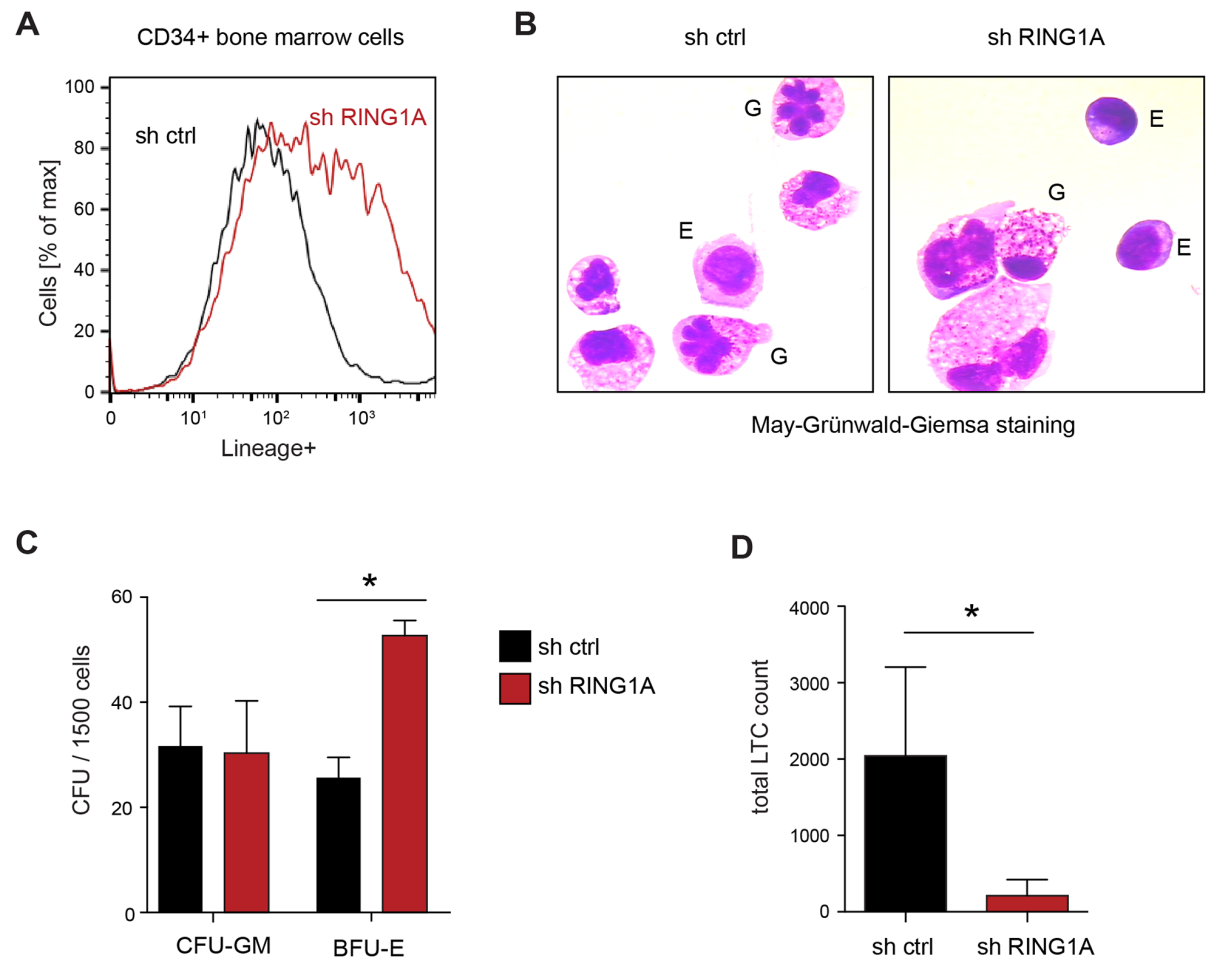
RING1A knockdown in primary healthy bone marrow CD34^+^ HSC **(A)** Percentage of lineage positive cells was measured by flow cytometry using of Lineage mix antibodies (CD4, CD8, CD15, CD19, CD41 and CD235alpha), comparing CD34^+^ cells transduced with control and shRING1A #1. **(B)** May-Grünwald-Giemsa staining of the same cells as A indicates both granulocytic (G) and erythroid (E) differentiation. A few examples are indicated. Erythroid cells show darker cytoplasm as a hallmark of hemoglobin synthesis. In particular during granulocytic differentiation, the ratio of cytosol to nucleus increases. **(C)** Colony forming assay in methylcellulose comparing control and shRING1A#1 transduced CD34^+^ cells. Data is represented as mean + SD (n = 3; ^*^, p < 0.05). CFU-GM, colony-forming-unit-granulocyte, macrophage; BFU-E, burst-forming-unit-erythroid. **(D)** Long-term culture colony-forming cells (LTC-CFC) of control and shRING1A#1 CD34^+^ cells after 6 weeks on stroma cell-supported culture. Data is represented as mean + SD (n = 4; ^*^, p < 0.05 paired, one-tailed *T*-test).

To further analyze the function of RING1A in hematopoiesis, we decided to analyze its role in primary HSPC. For this we enriched hematopoietic stem and progenitor cells by isolating CD34^+^ cells from the bone marrow from healthy donors. Using the same lentiviral knockdown strategy to suppress the expression of RING1A, we isolated transduced cells by sorting for the fluorescent marker GFP present on the vector. Analyzing these cells by flow cytometry after six days in culture, we observed an enrichment of cells positive for a mix of lineage markers compared to empty vector control cells suggesting spontaneous differentiation (Figure 4A). Cytologic analysis of these cells indicated that this included both granulocytic and erythroid differentiation (Figure 4B). To test whether the knockdown of RING1A would preferentially affect one of these lineages, we seeded the manipulated cells into methylcellulose and assessed the number and type of colonies. Specifically, we found that knockdown of RING1A significantly increased the number of erythroid colonies (BFU-E, units formed by erythroid progenitors), while leaving the number of granulocyte-macrophage colonies (CFU-GM) unaffected (Figure 4C). To test whether suppression of RING1A also affects LT-HSC, we assessed the colony forming capacity of manipulated CD34+ cells after 6 weeks in long-term culture on supportive stroma cells (Figure 4D). This assay provides a suitable approximation of the number of primitive LT-HSC. We observed that under these experimental conditions the number of colonies was markedly reduced in RING1A-depleted cell populations. Taken together, these results suggest that genetic RING1A inhibition in CD34^+^ cells leads to the depletion of the LT-HSC pool by increasing cellular differentiation, in particular towards the erythroid lineage.

### Pharmacological inhibition of RING1A depletes progenitors during induced erythroid differentiation

**Figure 5:**
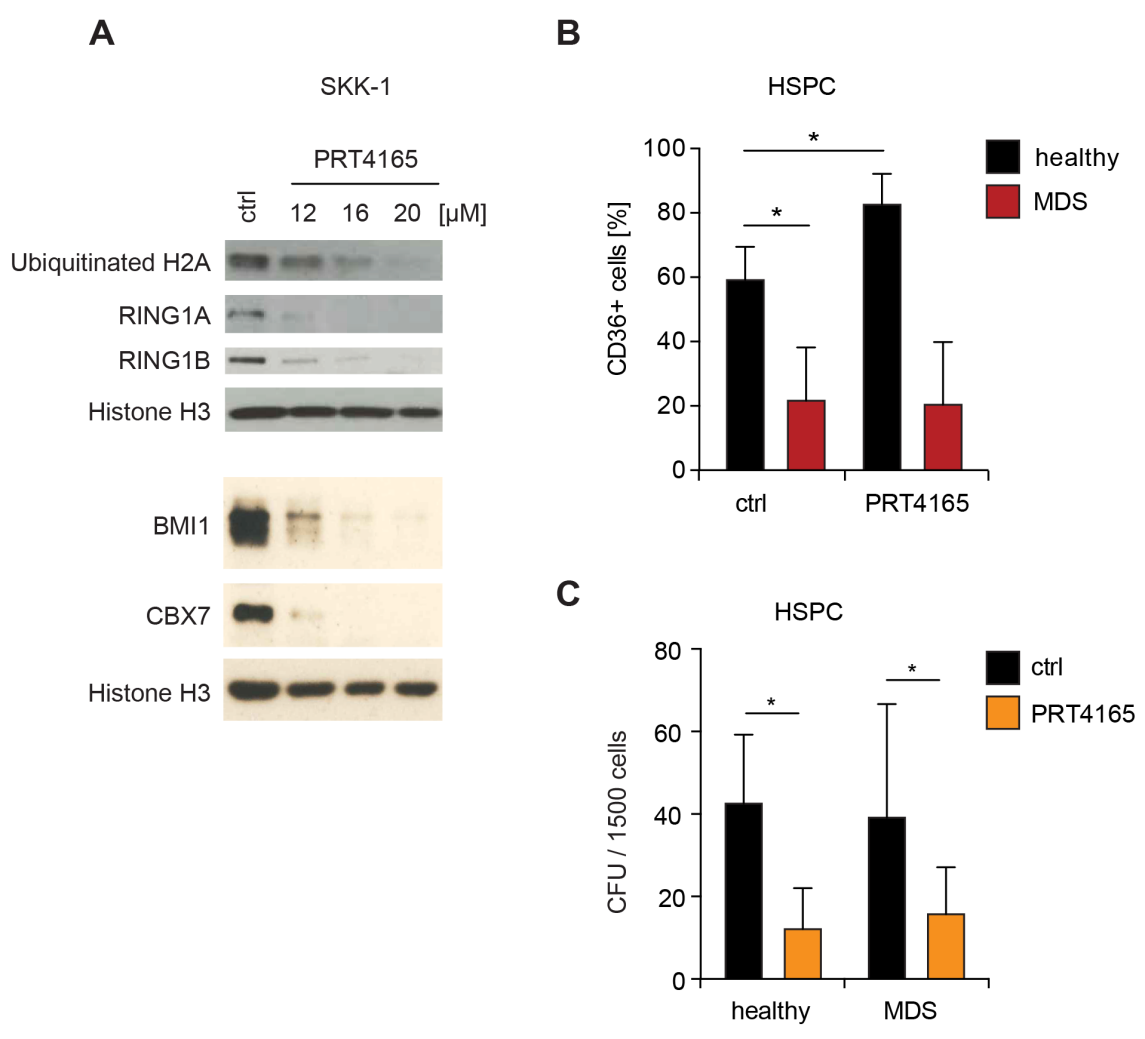
Differential influence of RING1 inhibitor PRT4165 in healthy and MDS cells **(A)** Western blot. To test the functionality of the RING1 inhibitor PRT4165, SKK-1 cells were treated with different concentrations or DMSO (ctrl) for six hours. Levels of PRC1 proteins and ubiquitinated H2A, the histone modification catalyzed by RING1 proteins, were analyzed by immunoblotting. Histone H3 served as loading control. **(B)** CD34^+^ HSPC from bone marrow of MDS patients (n=9) and healthy donors (n=4) were treated with 12.5μM PRT4165 or DMSO (control) for two weeks and with 2U/ml erythropoietin during the second week. The percentage of cells expressing the erythroid differentiation marker CD36 was assessed by flow cytometry. ^*^, p<0.05. **(C)** Same cells as in B were plated after treatment into methylcellulose and colonies were counted after 14 days of seeding. ^*^, p<0.05. CFU, colony-forming-unit.

The identification of novel therapeutic targets and the development of targeted strategies are urgently required to improve the management of MDS. Thus, we wondered whether pharmacological inhibition of RING1A has a therapeutic window for the treatment of MDS. Since RING1A is the enzymatically active subunit of PRC1 it is well suited for the inhibition with synthetic small compound inhibitors. Until now PRT4165 is the only reported small compound inhibitor of PRC1 and was shown to inhibit the H2A ubiquitin ligase activity of both RING1A and RING1B [31]. We first confirmed that the reported dose of PRT4165 was effective in SKK-1 cells and able to reduce the levels of ubiquitinated H2A and – possibly by inducing degradation – of PRC1 complex components (Figure 5A). In order to assess the potential of RING1A as drug target we compared the influence of PRT4165 treatment on MDS patient-derived CD34^+^ bone marrow cells with control HSPC from healthy donors. Specifically, we assessed differentiation capacity focusing on the erythroid lineage and the total number of colony-forming cells. For the differentiation analysis we analyzed the number of cells expressing the erythroid differentiation marker CD36^+^ after 14 days of culture with early-acting growth factors followed by induction with erythropoietin for 7 days. In healthy samples, on average, 60% of the cells were positive for CD36 and this number was significantly increased after co-administration of PRT4165 (Figure 5B). In contrast, in MDS patient samples the induction of CD36^+^ cells was severely impaired and not affected by PRT4165 treatment (Figure 5B). When plating erythropoietin-induced cells in methylcellulose the co-treatment with PRT4165 resulted in a depletion of colony-forming progenitor cells, which was comparable in MDS and healthy controls (Figure 5C). While these results further support a role of RING1A-containing PRC1 in the maintenance of stem and progenitor cells by limiting their differentiation, they also indicate that the only currently available inhibitor does not favorably discriminate healthy from diseased cells and cannot overcome the differentiation defect in MDS.

### RING1A expression is associated with number of cytopenias in MDS patients

**Figure 6:**
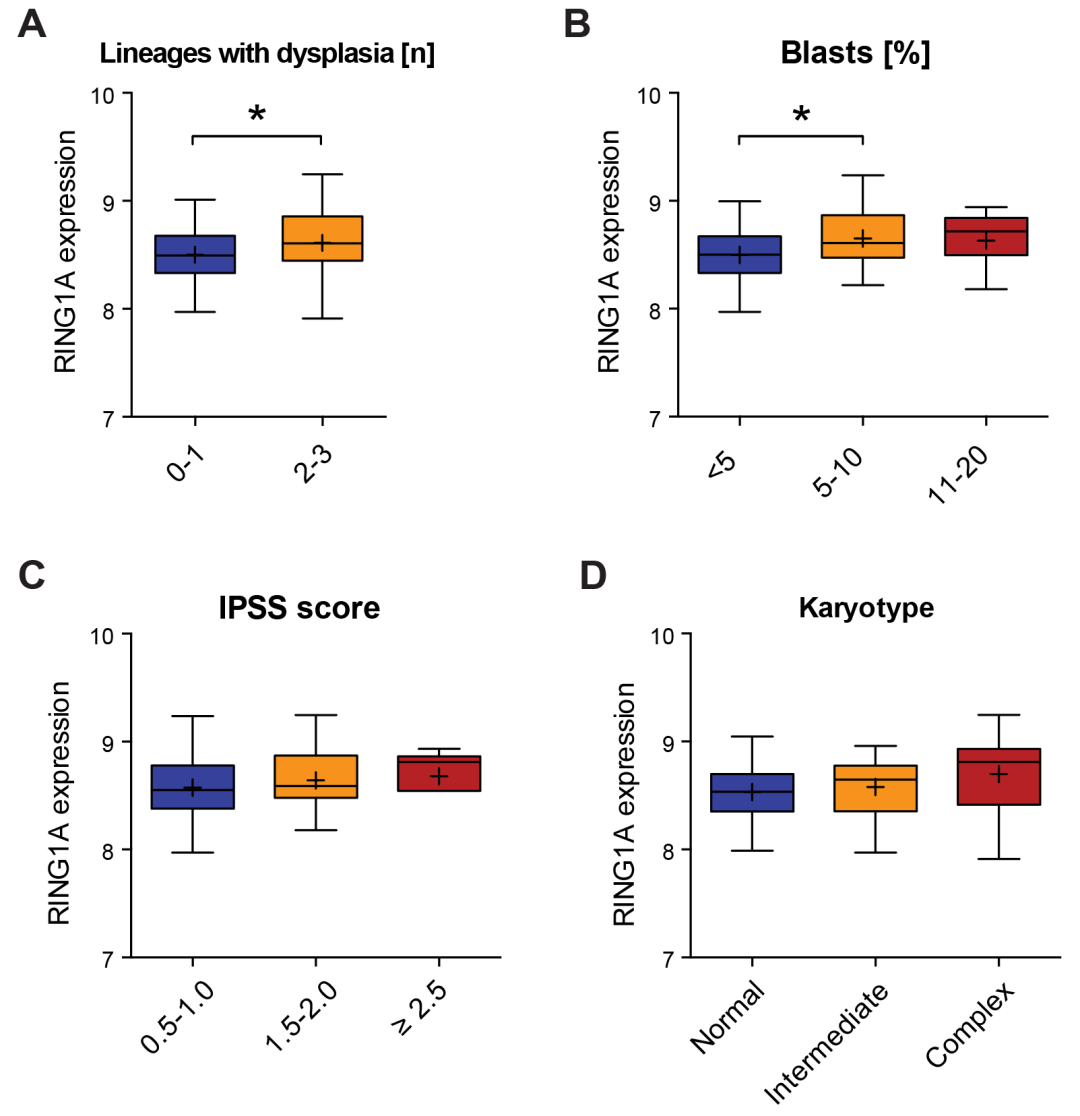
RING1A expression is associated with the number of dysplastic lineages **(A-D)** Boxplots showing the expression of RING1A in patients grouped according to clinical characteristics [32]. For the number of patients that were considered in each category please see Supplementary Table 1. The same probe that scored different in Figure 1A was analyzed and the binary logarithm of its intensity is plotted as an indirect measure of gene expression. ^*^, p < 0.05 using parametric unpaired T-test. A. RING1A expression according to number of lineages affected by dysplasia at diagnosis. B. RING1A expression according to percentage of blasts in bone marrow. C. RING1A expression according to IPSS scoring system [3]: 0.5 to 1.0, intermediate-1; 1.5 to 2.0, intermediate-2; and ≥2.5, high. D. RING1A expression according to karyotype with more than 3 alterations being considered as complex.

MDS is characterized by dysplasia in the bone marrow and cytopenia in the peripheral blood, affecting one or more lineages. Since RING1A limited differentiation of *in vitro* culture of both primary CD34^+^ cells and an established MDS/AML cell line, we wondered whether in MDS patients the levels of RING1A would correlate with the severity of the disease. We took advantage of previously generated expression data from mononuclear bone marrow cells from 139 MDS patients for which clinical data was well documented as part of the MILE study [32]. As shown in Figure 6A, the expression of RING1A was significantly increased in MDS patients displaying dysplasia in more than one lineage. In agreement with an earlier study [33], we found RING1A levels correlating with blast counts when comparing cases with low and intermediate counts (Figure 6B). In contrast to the same report, the differences in RING1A expression between different IPSS risk groups did not reach significance (Figure 6C). The expression level of RING1A was not associated with the type of chromosomal abnormalities (Figure 6D).

Our results indicate that the capacity of RING1A to inhibit differentiation *in vitro* is also reflected in MDS patients where higher RING1A levels correlated with a higher number of dysplastic lineages, and thus likely a higher number of cytopenias and greater severity of the disease. This suggests that the overexpression of RING1A in MDS may indeed be clinically relevant.

## DISCUSSION

### RING1A is a key PRC component in the hematopoietic stem cell compartment and MDS

In our study we have combined data mining of MDS patient cohorts and functional studies in primary cells and in an established MDS/AML cell line to investigate the genes encoding the components of PRC1. Taken together, our results suggest that RING1A is a key component of the PRC1 complex during normal hematopoiesis and in MDS. First, of the two enzymatic subunits of PRC1, RING1A is highest expressed in CD34^+^ HSC and further overexpressed in cells from MDS patients with RAEB-2. Second, during normal granulocytic differentiation RING1A is progressively downregulated. Third, inhibition of RING1A by RNA interference – but not inhibition of RING1B or other tested PRC1 genes – increased the differentiation of SKK-1 cells, an established MDS/AML cell line. Fourth, both genetic and chemical inhibition of RING1A increased the differentiation of healthy CD34^+^ bone marrow cells towards the erythroid lineage. Finally, in MDS patients higher RING1A expression was associated with a higher number of dysplastic lineages and blast counts > 5%.

Of note, van den Boom and colleagues previously assessed the function of various PRC1 subunits in human CD34^+^ cells isolated from cord blood [34]. They reported that when performing colony-forming assays, knockdown of several PRC1 components including the alternate catalytic subunit RING1B caused a shift towards the granulocytic-macrophage lineage in colony forming assays. Here, we have observed the opposite phenotype – a shift towards erythroid colonies - when knocking down RING1A. This suggests that differently composed PRC1 complexes might limit the commitment to specific blood lineages and conversely favor others. RING1A-containing PRC1 complexes seem to preferentially inhibit differentiation towards the erythroid lineage.

In our study, we have included CBX6 which we found upregulated in RAEB-2 patients. Knockdown of CBX6 promoted differentiation in SKK-1 cells albeit to a lesser extent than RING1A and only when treated with ATRA. However, several studies have shown that CBX6 preferentially associates with non-PRC proteins and thus questioned the role of CBX6 as canonical PRC1 component [35, 36]. In hepatocellular carcinoma CBX6 overexpression contributes to tumor progression and is predictive of a poor prognosis [37]. Thus, it will be interesting to continue the analysis of CBX6 in MDS although its role might not be related to PRC1 function.

Taken together, our results suggest that in normal hematopoiesis and MDS RING1A is a key component of PRC1 whose function is likely further modulated by its other components.

### Can PRC genes serve as prognostic markers?

Revised International Prognostic Scoring System (IPSS-R) has achieved international acceptance to estimate prognosis in MDS patients [4]. In addition to bone marrow blast percentage and the number of peripheral cytopenias, the IPSS-R is strongly based on cytogenetic information. A major challenge for the field is to further improve this prognostic scoring system by the incorporation of molecular information. The mutation status and the expression level of several PRC components and regulators have been found associated with disease stage, progression or response to treatment (recently reviewed in [38]). For instance, mutations in the PRC2 genes *EZH2* and *ASXL1* correlate with poor overall survival in MDS patients, independently of established risk factors, such as age, WHO classification or IPSS [18]. The levels of the PRC1 component BMI1 are associated with high-risk MDS with excess blasts and patients with lower risk MDS subtypes were more likely to progress when *BMI1* levels were high [39]. Analyzing a cohort of 54 MDS patients Xu and colleagues reported that the expression of BMI1, EZH2 and RING1A was higher in high-risk MDS compared to low-risk MDS and associated with increased IPSS score [33]. Analyzing larger cohorts we were able to confirm the overexpression of RING1A in MDS with excess blasts (Figure 1A) but found no significant association with IPSS scoring (Figure 6C). Specifically, both Xu *et al* and we observed higher RING1A expression in patients with 5-10% of blasts compared to those with less than 5% but could not observe any further increase when analyzing pre-leukemic patients with 11-20% blasts. In contrast to the same study but in accordance with another [40], we found EZH2 significantly downregulated in MDS RAEB-2 (Figure 1A) but also in subtypes associated with lower risk (Supplementary Figure 1A). The downregulation of EZH2 parallels the frequent inactivation of the gene by mutations [20, 21] and suggests that reduced PRC2 function is a more general hallmark of MDS than previously appreciated.

The expression of PRC genes is intimately linked to cell proliferation (discussed in [41]). First, proliferation-promoting signaling pathways upregulate the expression of some PRC genes. Second, PRCs promote proliferation through repressing the INK4-ARF locus, which encodes key cell cycle inhibitors. Thus, overexpression of PRC genes might be a consequence of an increased proliferation rate and less informative than direct markers of cell proliferation. Proliferation rates tend to decline with an advance in differentiation [22]. The finding that in particular PRC1 genes are upregulated during differentiation argues against an over-simplification of the relation between PRC gene expression and proliferation. More complex scenarios need to be considered in which changes in PRC1 composition allow them to perform cell-type and gene-specific functions.

In conclusion, the relation of PRC gene expression and MDS progression is complex as some PRC genes are upregulated in MDS while others are disrupted or downregulated. Future studies will need to carefully assess whether measuring mRNA levels and mutations status of PRC genes can add value to the current prognostic scoring.

### Is RING1A a drug target for MDS therapy?

Most chemotherapeutic agents target actively dividing cells. Thus, leukemic stem cells are relatively resistant to these kinds of drugs, contributing to treatment failure and ultimately patient death. Targeting pathways that are especially important for leukemic stem cells may offer a better therapeutic option for the patient [42]. Epigenetic therapy, that means strategies interfering with the chromatin regulation, has started to receive increasing interest [43]. DNA demethylating agents azacitidine and decitabine have been approved for the treatment of MDS [8] and the histone deacetylase inhibitor vorinostat is used for cutaneous T-cell lymphoma therapy [44]. Inhibitory compounds targeting components of PRCs are under development. In particular for PRC2′s enzymatically subunit EZH2, several inhibitors have shown promising pre-clinical results and now entered into clinical trials for solid cancers in which EZH2 is frequently overexpressed [45]. RING1A and RING1B provide the catalytic activity to PRC1 and are thus amenable for pharmacologic inhibition. Based on two observations we decided to evaluate the potential of RING1A as drug target for MDS. First, RING1A is overexpressed in MDS with excess blasts. Second, RNA interference mediated suppression of RING1A facilitated the differentiation of an MDS/AML cell line and of primary bone marrow HSPC (Figure 2 and 3). For this we used the only current available inhibitor PRT4165 [31]. Although the inhibitor is effective as shown by a pronounced reduction in RING1A-mediated ubiquitination of histone H2A, rather high and micromolar concentrations are required. Although PRT4165 is far from being a usable drug in the clinic, it serves as a tool compound and a lead structure for further synthetic improvement. When comparing the influence of PRT4165 on HSPC from MDS patients to healthy donors, we observed that it depleted the stem cell compartment in both MDS and healthy donor cells. While PRT4165 further increased erythropoietin-induced erythroid differentiation in healthy bone marrow cells, the influence of erythropoietin was severely impaired in MDS cells and not further affected by PRT4165 (Figure 5B and 5C) likely reflecting the complex differentiation defect, which is a hallmark of MDS. These results support a key role for RING1A in early hematopoiesis but also indicate that its pharmacologic inhibition has a limited therapeutic window as a single agent. When given in combination with a drug that discriminates between healthy and diseased stem cells, RING1A inhibition might have a more favorable enhancing effect. Others have obtained similar results testing the inhibition of BMI1, the PCGF component of PRC1 predominantly expressed in CD34^+^ bone marrow cells (Supplementary Figure 1B). Kreso and colleagues have used a compound that inhibits the BMI1 mRNA transcript by a yet unknown mechanism [46] which reduces the viability of both healthy and diseased bone marrow cells [47].

Chromosomal abnormalities involving gains and losses of gene copies are frequent in MDS [5]. Given the large number of PRC genes, these alterations frequently affect PRC genes. SKK-1 cells for instance have three copies of *PHC1* but only one copy of *EZH2* and *PCGF3* [26]. It will be interesting to test whether reduced dosages of key PRC components can be exploited for synthetic lethality approaches. It remains to be seen whether haploinsufficiency of SKK-1 cells for the catalytic PRC2 component EZH2 contributed to the sensitivity to PRC1 inhibition that we observed here.

Our and previous work show that PRC1 complexes containing RING1A, most likely together with BMI1, are emerging as key epigenetic regulators of differentiation in HSPC. The enzyme RING1A is amenable for pharmacologic inhibition but the therapeutic potential of inhibiting PRC1 will likely be limited to combinations with other disease-specific drugs.

## MATERIALS AND METHODS

### Antibodies and plasmids

The following antibodies were used for immunoblotting in a dilution of 1:500 (if not indicated otherwise): anti-RING1A (Abcam, ab32644), anti-RING1B (kindly provided by Luciano di Croce and described in [48]), anti-ubiquitinated H2A (Cell signaling, #8240), and anti-histone H3 (Abcam, ab1791, dilution 1:10.000). Conjugated antibodies used for flow cytometry are listed in Supplementary Information Supplementary Table 2.

ShRNAs have been inserted into the lentiviral SGEP vector following the described cloning protocol [28]. For a list of all used shRNAs please see Supplementary information Supplementary Table 3. Vectors encoding the modified CRISPR/SAM (Addgene) and the cloning protocol for the design and insertion of the guide strand have been described [29]. For the production of lentiviral particles we used packaging plasmids psPax2 (Addgene #12260) and pCMV-VSV-G (Addgene #8454).

### Cell culture

The MDS/AML SKK-1 cell line was obtained from the Leibniz-Institute DSMZ-German Collection of Microorganisms and Cell Cultures (Braunschweig, Germany) and cultured at 37°C in 5% CO_2_ in non-treated flasks for suspension cells using RPMI 1640 medium (Gibco). HEK293T cells were obtained from ATCC and cultured in DMEM. Media were supplemented with 10% fetal bovine serum (Gibco), 1% penicillin-streptomycin (Gibco) and 1% L-Glutamine (Gibco). Cells were authenticated and passaged for less than 6 months. To induce differentiation SKK-1 cells at an initial concentration of 0.5×10^6^ cells/mL were treated with 1μM ATRA (Sigma-Aldrich) for 2-4 days.

The AML Kasumi-1 cell line was obtained from ATCC and cultured at 37°C in 5% CO_2_ in non-treated flasks for suspension cells using RPMI 1640 medium (Gibco). Media was supplemented with 20% fetal bovine serum (Gibco), 1% penicillin-streptomycin (Gibco) and 1% GlutaMAX (Gibco). To induce differentiation, Kasumi-1 cells were treated with 200 nM PMA (Sigma-Aldrich) for 24 hours.

### Isolation and culture of CD34^+^ primary bone marrow cells

Ficoll separation was performed to isolate mononuclear cells from bone marrow samples obtained from MDS patients or healthy donors following informed consent and under institutional review board guidelines. CD34^+^ cells were isolated from mononuclear cells using magnetic beads CD34^+^ separation protocol (CD34 MicroBead Kit human, MACS Miltenyi Biotec). Isolated CD34^+^ bone marrow cells were kept in cell culture at 37°C in serum-free medium containing 20% BIT9500 (Stemcell Technologies), 80% IMDM medium with Glutamax (Gibco), 10μM ß-Mercaptoethanol (Gibco), 8μg/mL ciprofloxacin (Fresenius Kabi) and 5 growth factors: 100ng/mL stem cell factor, 100ng/mL FLT3-Ligand, 25ng/mL *thrombopoietin (*TPO), 10ng/mL interleukin 3 and 10ng/mL interleukin 6 (R&D systems).

### Gene transduction

Infections were essentially carried out as described [49]. In brief, viral supernatants were harvested from transfected HEK293T cells. Filtered lentiviral supernatants were mixed with cells at a final concentration of 0.5 × 10^6^ cells/mL in 6-well plates while centrifuging at 1200 rpm for 45 minutes at 37°C in the presence of 8μg/mL polybrene (Sigma-Aldrich). The infection process was repeated after 24 hours. For the infection of CD34^+^ primary cells we used retronectin-bound virus method (TaKaRa clontech), in which lentiviral particles are first bound to the plate coated with RetroNectin reagent, and the target cells are added after removing the virus supernatant. In this way, the viral supernatant was added to the Retronectin coated and blocked plates. The plate was centrifuged during 2 hours at 32°C 1000-2000g. The viral supernatant was removed and CD34^+^ primary cells were added to each well and centrifuged for 5 minutes to spin them down. Second infection hit was repeated after 24 hours. Infected CD34^+^ cells were cultured in serum-free medium as described above, 6 days after the first infection, GFP^+^ cells were sorted using FACSAria III cell sorter (Becton Dickinson). Infected SKK-1 were selected with 1 μg/mL puromycin (Sigma-Aldrich) 24 hours after the first infection.

Transient transfection of Kasumi-1 cells with a pool of 4 siRNAs against RING1A, RING1B or non-targeting control (GE Dharmacon) was performed with the electroporation-based Neon transfection system (ThermoFisher Scientific), following the suppliers’ instructions. For a list of siRNAs used please see Supplementary information Supplementary Table 3.

### Endogenous gene activation using CRISPR/SAM

For the activation of an endogenous gene locus, we have used CRISPR/Cas9 Synergistic Activation Mediator (CRISPR/SAM), an engineered protein complex described elsewhere [29]. Following authors’ protocol, cells were transduced with two lentiviral vectors (dCas9-VP64-blasticidin, Addgene #61425; and MS2-p65-HSF1-hygromycin, Addgene #61426) and selected with 2μg/mL blasticidin (Sigma-Aldrich) and 100μg/mL hygromycin B (Invitrogen) during 7 days. Once selected, a second lentiviral transduction was performed with a plasmid encoding a small guide RNA (sgRNA) of interest (Addgene #61427) into the cells stably expressing dCas9 and MS2. Finally, cells were selected with 25μg/mL zeocin (EMD Millipore) for 7 days and endogenous gene activation was analyzed by qRT-PCR or immunoblotting.

### RNA and protein analysis

The analysis of RNA and proteins was performed essentially as previously described [50].

### Flow cytometric analysis of differentiation

We assessed CD117 expression of infected SKK-1 cells by using PE Mouse anti-human CD117 antibody (BD Pharmingen) and we analyzed the CD117^+^ cells in the GFP^+^ population, in LSR Fortessa (Becton Dickinson) flow cytometer. A minimum of 10000 events of every sample were analyzed.

To assess the lineage commitment of primary CD34^+^ cells, we incubated them first with a mix of biotin-labeled antibodies of lineage markers that included CD4, CD8, CD15, CD19, CD235alpha and CD56 antibodies. Fluorophor-couped streptavidin was added. After washing, cells were analyzed in HF2 buffer (HBSS 1x, 2% FCS (Biochrom), 10mM Hepes, 1% penicillin-streptomycin) containing 1μg/mL propidium iodide (PI) (Sigma-Aldrich) to exclude PI^+^ dead cells, were analyzed by flow cytometry (CyAn Beckman coulter cytometer). For analysis of erythroid differentiation, cells were labeled with anti-CD36. For a detailed list of all antibodies, sources and dilutions please see Supplementary Info Supplementary Table 2. Analysis was performed using FlowJo^TM^ software.

### Colony formation

1500 primary CD34^+^ cells were plated into methylcellulose. For this, cells were first diluted in 300μL serum-free medium (adding inhibitor or vehicle if indicated) and then added to 3000μL of methylcellulose (StemMACS HSC-CFU complete with Epo, 130-091-280, Miltenyi Biotech) and divided into 2 plates. Dishes were incubated at 37°C and colonies were counted and classified after 14 days using standard criteria.

### Long-term culture assay

At day 0, EL08-1D2 murine stroma cells were plated in 6-well plates (0.4×10^6^ cells/well) in stroma medium (80% Alpha MEM medium (Gibco), 15% FCS (Biochrom), 5% horse serum (Stemcell technologies), 1% penicillin-streptomycin and 10μM β-mercaptoethanol). At day 2, when cells were confluent, they were irradiated (30 Gy) and media was changed for fresh medium. At day 9, medium was removed and 10000 to 20000 GFP^+^ CD34+ cells (control or shRING1A) were added in LTC-medium (2mL/well), containing 50mL Myelocult medium (H5100 #05100, Stemcell technologies), 10ng/mL FLT3-ligand (R&D systems), 20ng/mL TPO (R&D systems), 1μM hydrocortisone (Pfizer), 8μg/mL ciprofloxacin (Fresenius Kabi) and 1% L-glutamine (Gibco).

Half of the media was replaced every week (once a week) with fresh media for 6 weeks, carefully removing 800μL supernatant and adding 1mL of fresh medium. After 6 weeks, total cells were counted and 1500 cells were plated into methylcellulose for CFU assay as described above and colonies were counted after 14 days using standard criteria.

### Chemical inhibition of RING1A and erythropoietin-induced differentiation

Toxicity tests were performed to determine the tolerated concentration of the RING1 inhibitor PRT4165 (Sigma-Aldrich). To assess an influence on erythroid differentiation, CD34^+^ bone marrow cells cultured as described above were treated with the well-tolerated concentrations of 12.5μM PRT4165 or DMSO for two weeks and with 2 U/ml erythropoietin during the second week. Then, cells were counted and split. 1500 cells were plated into methylcellulose and the remaining viable cells were analyzed by flow cytometry.

### May-Grünwald-Giemsa staining

Cells were immobilized on glass slides by cytospin centrifugation (Thermo Scientific) at 300 rpm for 10 minutes. Once dried, cells were submerged in fixation May-Grünwald solution (Merck) for 5 minutes followed by 15 minutes in staining Giemsa solution (Merck). Cells were mounted with DPX Mountant for histology (Sigma-Aldrich).

### High content data analysis

All data analysis was performed using publicly available datasets. The MDS datasets GSE4619 [23] or GSE19429 [24] were generated using cDNA from CD34^+^ cells from 55 or 183 MDS patients followed by microarray analysis using the Affymetrix GeneChip Human Genome U133 Plus 2.0 arrays. The arrays were normalized using Robust Multi-Array Average expression measure (RMA) and differential expression analysis performed using a linear model and the Limma package [51]. Moderated t-statistics were generated and significance was assessed using log fold change and P-value.

To analyze the differentiation from HSC to fully mature polymorphonuclear granulocytes the GEO series GSE42519 was used, which consists of data from isolated bone marrow cell populations that represent eight sequential stages in the differentiation from HSC to fully mature polymorphonuclear granulocytes [25]. The normalized expression values were extracted and unsupervised hierarchical clustering performed using the heatmap.2 function from gplots (https://cran.r-project.org/package=gplots).

In order to compare RNA expression and MDS clinical features, the dataset GSE15061 was utilized [32]. This dataset consists of expression data from mononuclear bone marrow cells from 139 MDS patients generated using the Affymetrix GeneChip Human Genome U133 Plus 2.0 array. The normalized expression values were extracted and correlation analysis performed using the R core package (http://www.R-project.org). Parameters considered for the correlation analysis were number of cytopenias at diagnosis, percentage of blasts in bone marrow and scoring according to IPSS.

### Statistics

If not indicated otherwise, p-values are calculated using paired, two-tailed T-tests.

## SUPPLEMENTARY FIGURES AND TABLES


