# Supplementary material for: Polycomb protein RING1A limits hematopoietic differentiation in myelodysplastic syndromes
Source: Oncotarget. 2017 Dec 1;8(70):115002–17. doi: 10.18632/oncotarget.22839 (PMC5777749; doi:10.18632/oncotarget.22839)
Supplement: Supplementary file 1 [file oncotarget-08-115002-s001.pdf]

Polycomb protein RING1A limits hematopoietic differentiation in myelodysplastic syndromes

SUPPLEMENTARY MATERIALS

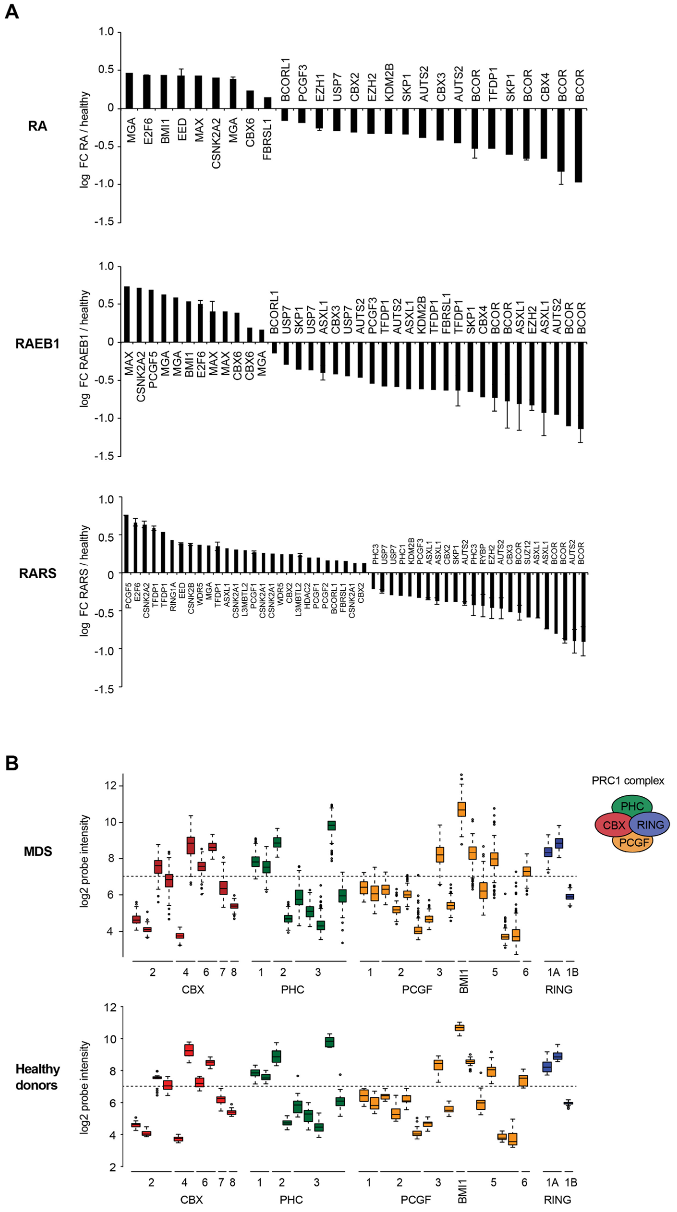

Supplementary Figure 1: Deregulated expression of PRC genes on MDS. (A) Comparison of PRC genes between RAEB-1, RARS, RA and healthy controls. Logarithmic fold change in expression of probes for canonical PRC genes and components of non-canonical complexes in MDS subtypes compared to healthy controls. Two datasets [23, 24] were analyzed and only significant fold-changes (FC, p-value < 0.05) are shown. When significant in both datasets, the mean is plotted and the variation indicated by error bars. **(B)** Boxplot showing microarray probes of canonical PRC1 transcripts in CD34^{+} BM cells from MDS patients and healthy donors. To check the expression of PRC1 components we have re-analyzed microarray-based expression data generated with cDNA from CD34^{+} cells from 183 MDS patients and 17 healthy donors [24]. The boxplot shows the probe intensities. We considered a gene expressed if the binary logarithm of the intensity of at least one of its probes was above 7. This indirect expression analysis suggests that *RING1A* and *BMI1 (PCGF4)* were the most prominently expressed RING1 and PCGF components, respectively.

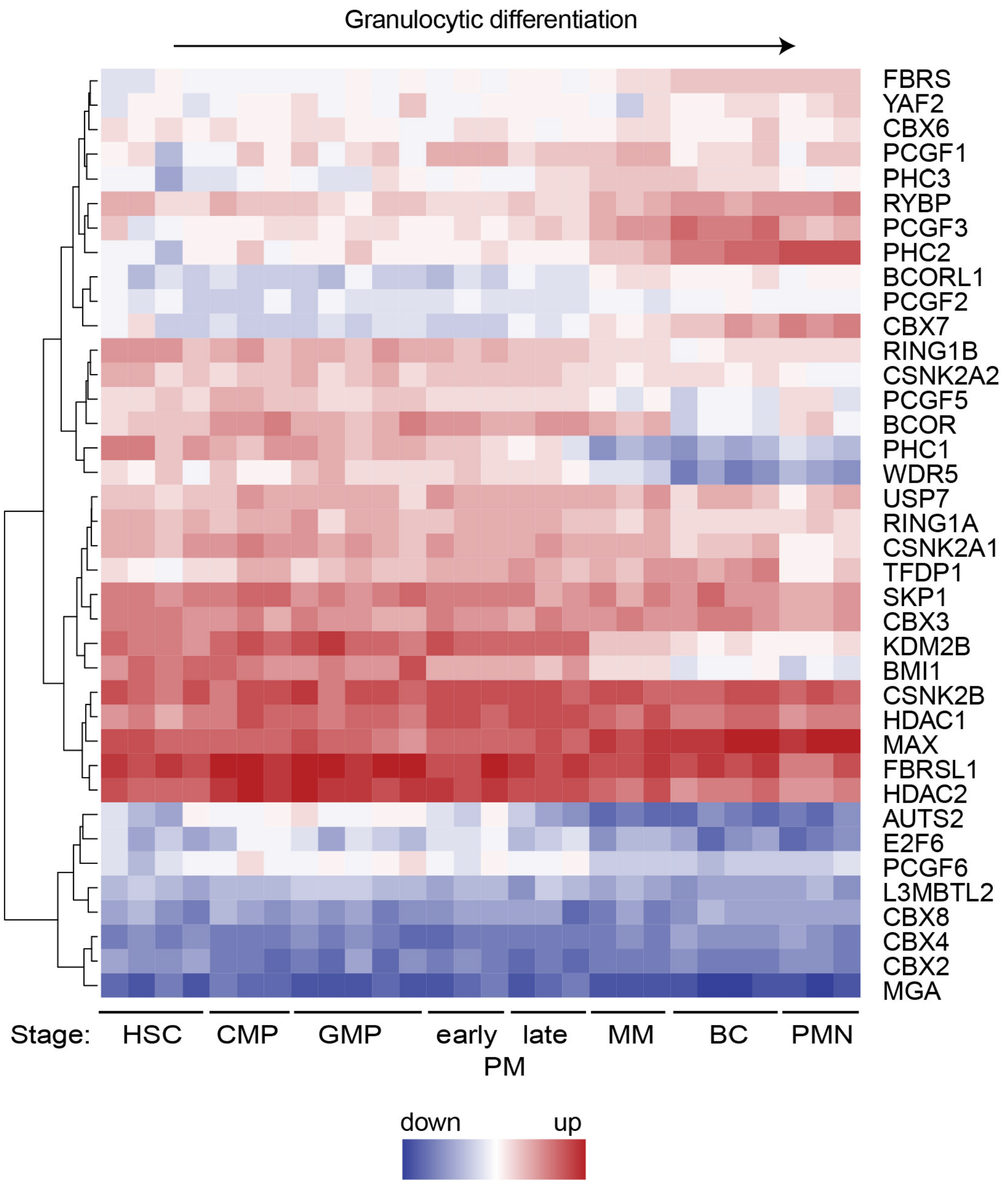

Supplementary Figure 2: Dynamic expression of PRC1 genes during granulocytic differentiation. Same heatmap as in Figure 1B including also genes encoding components of non-canonical PRC1 complexes and PRC2.

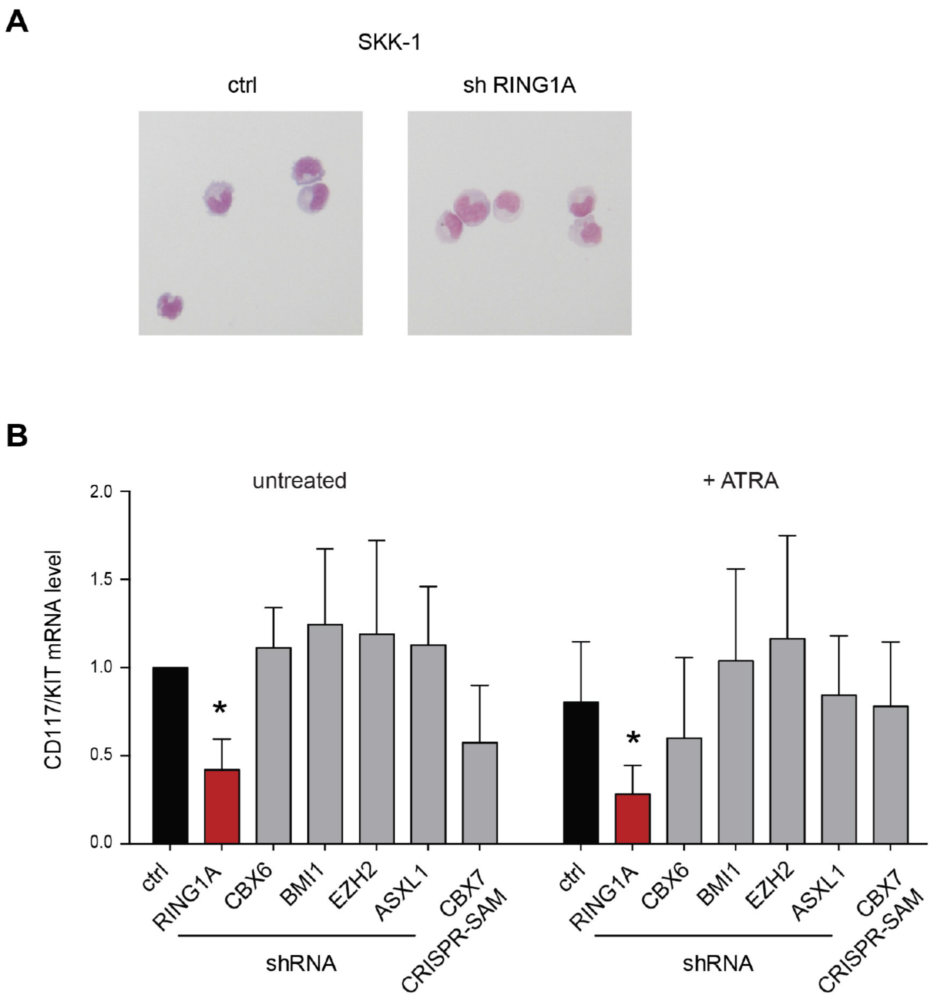

Supplementary Figure 3: Cytology and CD117 expression of SKK-1 cells. (A) Control and shRING1A SKK-1 cells treated with 1 µM ATRA for 4 days observed after May-Grünwald-Giemsa staining. SKK-1 shRING1A cells showed reduced basophilic staining compared to control cells. **(B)** Genetically perturbed SKK-1 cells characterized in Figure 2B and C were treated with 1 µM ATRA for 2 days and mRNA of the CD117-encoding KIT gene were measured by qRT-PCR. Data is represented as mean + SD ($n \geq 4$; *, p-value < 0.05 compared to control set to 1).

Supplementary Table 1: Data from MILE study used for Figure 6

RING1A Exp. Level[a]	Dysplasia [n]		Blast score			Karyotype			IPSS		
	0-1	2 or 3	< 5	5-10	10-20	Normal	Intermediate	Complex	0.5-1.0	1.5-2.0	>= 2.5
**Low**	68.3 (41 of 60)	31.7 (19 of 60)	76.7 (46 of 60)	13.3 (8 of 60)	10 (6 of 60)	86.7 (52 of 60)	8.3 (5 of 60)	5 (3 of 60)	69(20 of 29)	27.6 (8 of 29)	3.4 (1 of 29)
**High**	48.1 (38 of 79)	51.9 (41 of 79)	58.2 (46 of 79)	22.8 (18 of 79)	19 (15 of 79)	79.7 (63 of 79)	11.4 (9 of 79)	8.9 (7 of 79)	59.6 (31 of 52)	30.8 (16 of 52)	9.6 (5 of 52)

[a]Low and high is defined as lower or higher than 8.5 of log2 of intensity, respectively, and referring to the probe that we identified significantly deregulated in MDS with excess blasts 2 patients (Figure 1A).

Supplementary Table 2: Antibodies for flow cytometry

Antibodies	Source	Catalog number	Dilution
CD34-perCP/Cy5.5 (clone 581)	Biolegend	343522	1:50
CD4 Biotin (clone RPA-T4)	Biolegend	300504	1:200
CD8 Biotin (clone RPA-T8)	Biolegend	301004	1:200
CD15 Biotin (clone W6D3)	Biolegend	323016	1:100
CD19 Biotin (clone HIB19)	Biolegend	302204	1:200
CD235alpha Biotin (clone HIR2)	Biolegend	13-9987-82	1:200
CD56 Biotin	BD Pharmingen	555515	1:200
CD45-RA-PECy7 (clone HI100)	BD Pharmingen	560675	1:20
CD123-Pacific Blue (clone 6H6)	Biolegend	306018	1:20
CD38-APC (clone HB-7)	BD Pharmingen	345807	1:40
Streptavidin-APC-Cy7	Biolegend	405208	1:100
CD36-APC-Cy7 (clone 5271)	Biolegend	336213	1:50
CD45-APC	eBioscience	17945942	1:100
CD235alpha-Pacific Blue	Biolegend	349108	1:50
CD105-FITC	Ancell	213510	1:20

Supplementary Table 3: shRNA and siRNA sequences

**shRNA**	**97mer oligo (for SGEP)**
RING1A #1 (shRING1A_1512)	TGCTGTTGACAGTGAGCGACCAGCCAATAAGAGGACACAATAGTGAAGCC ACAGATGTATTGTGTCCTCTTATTGGCTGGCTGCCTACTGCCTCGGA
RING1A #2 (shRING1A_675)	TGCTGTTGACAGTGAGCGACAGGGTCAGATCAGACCACAATAGTGAAGCC ACAGATGTATTGTGGTCTGATCTGACCCTGGTGCCTACTGCCTCGGA
RING1B #1 (shRING1B_619)	TGCTGTTGACAGTGAGCGACAGGATCAACAAGCACAATAATAGTGAAGCC ACAGATGTATTATTGTGCTTGTTGATCCTGGTGCCTACTGCCTCGGA
BMI1 #1 (shBMI1_1673)	TGCTGTTGACAGTGAGCGCAAGATTGTTGTTATAAAGAATTAGTGAAGCCA CAGATGTAATTCTTTATAACAACAATCTTTTGCCTACTGCCTCGGA
BMI1 #2 (shBMI1_1810)	TGCTGTTGACAGTGAGCGCTAGTTTGTTAATCTCAACTAATAGTGAAGCCA CAGATGTATTAGTTGAGATTAACAAACTATTGCCTACTGCCTCGGA
CBX6 #1 (shCBX6_1190)	TGCTGTTGACAGTGAGCGCCCTCCTGACGGTCACAATCAATAGTGAAGCC ACAGATGTATTGATTGTGACCGTCAGGAGGTTGCCTACTGCCTCGGA
CBX6 #2 (shCBX6_578)	TGCTGTTGACAGTGAGCGCCCGCATCATCCTGAACCTGAATAGTGAAGCCA CAGATGTATTCAGGTTCAGGATGATGCGGTTGCCTACTGCCTCGGA
ASXL1 #1 (shASXL1_431)	TGCTGTTGACAGTGAGCGAAAGGATGAAGGACAAACAGAATAGTGAAGC CACAGATGTATTCTGTTTGTCCTTCATCCTTCTGCCTACTGCCTCGGA
ASXL1 #2 (shASXL1_485)	TGCTGTTGACAGTGAGCGAGCGCCTGGTATTAGAAAACTATAGTGAAGCC ACAGATGTATAGTTTTCTAATACCAGGCGCGTGCCTACTGCCTCGGA
EZH2 #1 (shEZH2_290)	TGCTGTTGACAGTGAGCGCCAGGATGGTACTTTCATTGAATAGTGAAGCCA CAGATGTATTCAATGAAAGTACCATCCTGATGCCTACTGCCTCGGA
EZH2 #2 (shEZH2_576)	TGCTGTTGACAGTGAGCGCCAGCAGAAGAACTAAAGGAAATAGTGAAGC CACAGATGTATTTCCTTTAGTTCTTCTGCTGTTGCCTACTGCCTCGG

**siRNAs**			
RING1A: ON-TARGETplus SMARTPOOL Human RING1 (6015) siRNA #L-006554-00 (GE Dharmacon)	J-006554-07	Target sequence:	CGAGGUAUGUGAAGACAAC
		Antisense:	GUUGUCUUCACAUACCUCG
	J-006554-08	Target sequence:	CUGGAGGGCGUCAGUGAAA
		Antisense:	UUUCACUGACGCCCUCCAG
	J-006554-09	Target sequence:	GAACUGAGUCUGUAUGAGC
		Antisense:	GCUCAUACAGACUCAGUUC
	J-006554-10	Target sequence:	UCUCUAAGAUCUAUCCUAG
		Antisense:	CUAGGAUAGAUCUUAGAGA
RING1B: ON-TARGETplus SMARTPOOL Human RNF2 (6045) siRNA #L-006556-00 (GE Dharmacon)	J-006556-05	Target sequence:	CGAGAUACAUAAAGACUUC
		Antisense:	GAAGUCUUUAUGUAUCUCG
	J-006556-06	Target sequence:	GUAUCUGGCUGUGAGGUUA
		Antisense:	UAACCUCACAGCCAGAUAC
	J-006556-07	Target sequence:	GGCAAUUGAUCCAGUAAUG
		Antisense:	CAUUACUGGAUCAAUUGCC
	J-006556-08	Target sequence:	ACAAAGGAGUGUUUACAUC
		Antisense:	GAUGUAAACACUCCUUUGU
